# Comparing cytocompatibility of two fluoride-containing solutions and two resin-based restorative materials—a pilot study

**DOI:** 10.3389/froh.2024.1330944

**Published:** 2024-04-08

**Authors:** Riaan Mulder, Naeemah Noordien, Nicoline Potgieter

**Affiliations:** ^1^ Department of Prothodontic Dentistry, University of the Western Cape, Cape Town, South Africa; ^2^ Department of Orthodontics and Paediatric Dentistry, University of the Western Cape, Cape Town, South Africa

**Keywords:** silver diamine fluoride, silver fluoride, resin restorative material, buccal fibroblast cells, resin modified glass ionomer

## Abstract

**Background:**

Cytocompatibility should always be considered, especially if the surface of treated carious lesions is close to soft tissue or is accidentally exposed to the oral soft tissue by the clinician.

**Methods:**

The aim of the present study was to compare the cytocompatibility of two fluoride-containing liquids and two resin-containing restorative materials with buccal mucosa fibroblasts. The fluoride-containing materials were silver diamine fluoride and water-based silver fluoride.

**Results:**

The statistical analysis was completed by comparing the positive control growth of the buccal mucosa fibroblasts to the growth of cells exposed to various materials. The one-way ANOVA with Tukey’s HSD result was completed. All the assessed materials compared to the control wells for both the 24 and 48 h time intervals indicated a significant cytocompatibility result, except for the test wells with Stela (SDI) at the 24 h time interval. There was no significant difference between the step 2 liquids and the two dental materials in cytocompatibility at the 24 h interval. All four materials indicated no significant differences between the cytocompatibility of any dental materials for 48 h.

**Conclusion:**

The cytocompatibility assessment for Riva Star and Riva Star Aqua with the direct method in a full dispensing drop is not viable for step 1 of the fluoride-containing liquids. The use of Stela Light Cure is a suitable material that will be in contact with buccal mucosa as it showed potential for increased cytocompatibility compared to Riva Light Cure. Riva Star Aqua is more cytocompatible than Riva Star.

## 1 Introduction

The use of silver diamine fluoride (SDF) has been well established for preventing the progression of dental caries and has shown promise in vulnerable populations as well as an intervention to caries progression (1). The ease of use is useful in treating large numbers of patients as a treatment modality for the prevention of caries (2). Various formulations of SDF have been assessed in the literature for caries remineralization, and the subsequent acid resistance of treated teeth (3) improved changes in the surface’s micro-hardness (4, 5); the management of carious lesions as a result of molar incisor hypomineralization have been recorded (6). It has been realized that teeth usually treated with various SDF products have large active cavitated carious lesions, with the tooth very well at the end of its life span. SDF products are, in most cases, the last chance for the survival of the tooth pulp. Some teeth might additionally receive a restoration that would inevitably have prolonged contact with the buccal mucosa; hence we have included two resin-based restorative materials for their cytocompatibility to buccal fibroblasts. Very deep pre-molar cavities of an *in vivo* study showed that Riva Light Cure (Southern Dental Industries (SDI) Limited, Australia) produced more damage to the pulp than Riva Self Cure Glass Ionomer (7). The *in vitro* material cytocompatibility results on pulp stem cells for Riva Light Cure indicated a significantly lower cytocompatibility to the pulpal stem cells compared to the control cells for all time intervals (24, 48, and 72 h of exposure) (8). However, these materials are deemed to be biocompatible (7) and are therefore the ideal comparative control materials for the assessment of cytocompatibility.

Initially, with the advent of SDF, pulpal cytocompatibility was debated (9, 10). The SDF products are not indicated for direct pulp application nor teeth with clinical signs of pulp involvement (11). However, the proximity of the carious lesion to the pulp cannot be ignored, hence the various cytocompatibility assessments that have been completed in the literature. The translation of *in vitro* studies toward the clinical sphere is represented by a clinical trial with a similar incidence of pulpal reactions in the patient groups for both the primary and secondary SDF-treated teeth groups (12). This pilot study is more focused toward the surrounding soft tissue of the oral cavity represented by buccal fibroblasts. In the initial stages of SDF use, it was predicted that oral tissue upon contact would present with reversible oral lesions (13). The importance of the buccal fibroblast cytocompatibility with SDF (Riva Star, SDI Limited, Australia) and water-based silver fluoride (AgF; Riva Star Aqua, SDI, Australia) gives further insight into the effect of these materials when they inadvertently come into contact with the oral soft tissue. In the literature, diluted versions of SDF showed inhibitory effects on cultured gingival fibroblasts with dilutions of 1,000 and 10,000 times (14). The clinician, however, uses an application brush and the volume of liquid is much bigger than the large dilutions used for *in vitro* studies. During SDF use and inadvertent contact with the soft tissue, the clinician will immediately start rinsing the affected area to reduce the tissue interaction and contact time. This technique was performed in a clinical trial and 3 of the 373 patients presented with small, mildly painful white lesions on the mucosa. There was complete resolution in 48 h (12). In another trial of 888 children, gum bleaching occurred in 38 children as a result of contact with SDF and resolved within 2 days (15). *In vitro* research has shown that there was a pH-induced chemical burn or at the very minimum mucosal corrosion with both 3 min and 1 h exposures of epithelial simulations. The authors also indicated that SDF was still less damaging than phosphoric acid etchant (16), which is widely used. SDF temporarily stains the skin as it does not penetrate the dermis; however, desquamation of skin results in skin pigmentation after 14 days with the shedding of keratinocytes (17, 18). The aim of this pilot study was to assess the long-term exposure, e.g., 24 and 48 h intervals, of these materials on buccal fibroblasts.

The use of the MTT [3-(4,5-dimethylthiazol-2-yl)-2,5 diphenyl tetrazolium bromide] assay provides a sensitive and quantitative colorimetric assay that measures the proliferation and viability of the cells’ mitochondrial activity based on the conversion of the MTT reagent into formazan crystals. These assessments of cell viability provide insight to potential cellular interaction; in particular to the buccal mucosa, simulated by buccal fibroblasts. This pilot study present results with a clinically relevant volume of a single drop from each step of the SDF and AgF bottles. The two formulations, namely SDF (Riva Star, SDI, Australia) and AgF (Riva Star Aqua, SDI, Australia), were assessed previously by the authors of this pilot study for the penetration of materials into carious tooth structure toward affected dentine. The neutral pH of AgF also resulted in remineralization, and a depth of ion penetration was found to be similar to that of SDF (Riva Star). It was also noted that the surface of the carious lesion exposed to the oral environment and inevitably the buccal mucosa has the highest concentrations of ions derived from the SDF and AgF materials (19). With the current SDF literature only utilizing microtiter volumes (14), a full dispensing drop would be used in this pilot study as per the volume dispensed from the dispenser bottle. This study hypothesized that the cytocompatibility of the caries remineralization products of SDF and AgF would be no different to the restorative materials that contact the buccal mucosal fibroblasts. The aim of this study was to compare the cytocompatibility with buccal mucosa fibroblasts directly exposed to SDF, AgF, and two control materials, namely Riva Light Cure (SDI) and Stela Light Cure (SDI).

## 2 Materials and methods

### 2.1 Sample preparation

Five restorative material samples (height of 1 mm and diameter of 3 mm) were produced according to the manufacturer’s recommendations for Stela (SDI) and Riva Light Cure (SDI). Two time periods would be assessed for the direct exposure of the restorative materials to the buccal mucosa fibroblasts in Dulbecco's Modified Eagle's Medium (DMEM), namely 24 and 48 h. The restorative material samples were sterilized with ethylene gas (Steri-Vac 4XL gas sterilizer, Model 400DGP; 3M Center, St Paul, MN, USA) stored at room temperature. The samples were assessed after 72 h to ensure the ethylene gas would not influence the cytocompatibility assay. The prepared restorative sample was placed directly in the well for direct contact with the medium and buccal fibroblast cells. Riva Star (SDF, SDI) and Riva Star Aqua (AgF, SDI) were used in their respective step 1 and 2 formulations to the volume of one dispensed drop, as that would be the clinical scenario. Each drop from the dispensing bottle (approximately 30 µl) (20) was placed directly into each well in the well plate.

### 2.2 Cytocompatibility assay

The cell viability assays were completed following an established method (21). This human oral fibroblast cell line was established in the Oral and Dental Research Institute, University of the Western Cape, as these fibroblasts were well suited to simulate the cells that would be exposed to the Riva Star and Aqua formulations as well as the restorative materials for the oral environment. Stocks of these cells were kept frozen in liquid nitrogen and retrieved for use. Cells were maintained and cultured in standard conditions. The viability of cells after the 24 and 48 h intervals of exposure was evaluated using the MTT assay.

To test the cytocompatibility of the products toward these buccal mucosa fibroblasts, the cells were first grown to near confluence and then were seeded in 96-well microtiter plates at a density of 1 ×10^5^ cells per well. The cells were maintained in DMEM containing 10% fetal bovine serum (FBS) and 1% penicillin-streptomycin cocktail (penstrep) HyClone™^;^ Cytiva, Marlborough, MA, USA) in a 37°C humidified incubator with 5% CO₂ saturation. After 24 h cells, were microscopically checked for strong cell growth and the culture medium was replaced with fresh medium containing the different samples (22). Two 96-well plates were produced with samples for a 24 and 48 h assessment period for cytocompatibility. The sample distribution in each 96-well plate therefore consisted of each well containing one material sample per time (e.g., SDF bottle 1: *n* = 5; SDF bottle 2: *n* = 5; AGF bottle 1: *n* = 5; AGF bottle 2: *n* = 5; Rival Light Cure: *n* = 5; and Stela Light Cure: *n* = 5) as well as 20 untreated control wells containing only buccal mucosa fibroblasts.

Cells were exposed to the sample/medium mix for 24 and 48 h after which the MTT assay was completed (21). A total of 100 μl of the MTT reagent [prepared from 5.0 mg/ml stock solution and diluted with DMEM medium using a dilution factor of 1:10 (Sigma)] was added to each well. The plates were incubated again at 37°C for 4 h. The MTT reagent was then removed and replaced with 100 μl alkaline dimethyl sulfoxide to dissolve the purple formazan crystals (23). The restorative material samples remaining in the wells were removed at this point. After a 15 min incubation period at 37°C, the absorbance of the samples was measured at 540 nm using the microtiter plate reader. The absorbance at 630 nm was used as a reference wavelength (SPECTROstar Nano; BMG LABTECH) (21). The percentage of cell viability was calculated to the control wells. Control values were taken as 100% of the average control-wells values and subsequently expressed as a percentage of 100% using the following formula: [100/(Optical density of the control buccal fibroblast cells × Optical density of the fibroblast cells exposed to the test materials)] – 100.

## 3 Results

The statistical analysis was completed by comparing the control growth as 100% of the buccal mucosa fibroblasts to the growth of cells exposed to the various materials. The one-way ANOVA with Tukey’s HSD result was completed. A *p*-value <0.05 indicated that significant differences were present. In the 96-well plate, the addition of the drop in step 1 from the Riva Star and Riva Star Aqua bottles resulted in an immediate change in medium color. This is indicative of a pH change in the medium. This resulted in a shock of the cells; upon completion of the cell culture, the wells were black and could not be read by the spectrophotometer. However, step 2 of both SDI products did not show a change in color in the wells, indicating that the change in pH only occurred with step 1 of both the SDF and AgF liquids.

Regarding the cells of the control wells, both the 24 and 48 h time intervals indicated a significant difference between the fluoride-containing liquids and the dental materials assessed, except for the test wells with the dental material Stela at the 24 h time interval. The comparison between both the step 2 liquids and the two dental materials showed that no significant difference in cytocompatibility was present at both the 24 and 48 h intervals (Table 1). Cytocompatibility improved from the 24 h interval to the 48 h interval and indicated that the Riva Star Aqua step 2 (42.74%) is comparable to Stela Light Cure at 53.09%. Riva Light Cure decreased in cytocompatibility from the 24 to the 48 h time interval.

**Table 1:** Cytocompatibility survival rate at different time intervals in percentage of the control wells.

	Riva Star step 1	Riva Star step 2	Riva Star Aqua step 1	Riva Star Aqua step 2	Riva Light Cure	Stela Light Cure
24 h	N/A	16.9	N/A	19.7	46.18	53.09
48 h	N/A	26.08	N/A	42.74	27.41	57.66

## 4 Discussion

The hypothesis of this study was accepted since the cytocompatibility of the caries remineralization SDF and AgF products were not significantly different to the biocompatible restorative materials in contact with the buccal mucosal fibroblasts.

The water-based Riva Star Aqua (AgF) uses water as the suspension medium instead of ammonia (SDF); otherwise, the formulation to Riva Star is similar to fluoride (5%), silver (25%), and ammonium iodide (8%). The difference became visible in the 48 h results where the cytocompatibility of the AgF (42.74) improved compared to SDF (26.08). This pilot study specifically focused on the effect of the SDF and AgF that would be in contact with the buccal mucosa, hence the use of the fibroblasts. Based on the comparison and the result showing no statistical significance, the SDF and AgF materials can be considered biocompatible. In clinical trials, a very small percentage of participants experienced oral lesions (12) and gum bleaching (15), further supporting the low incidence when the products are handled correctly.

There is a clear transition of ions from the surface of the treated tooth toward the pulpal cells. The weight percentage of ions decreases as the SDF and AgF move through the tooth structures (19). Step 1 for both materials could not be assessed for buccal fibroblast compatibility due to the silver precipitation of the material. In the literature, step 1 of Riva Star was assessed in a study of SDF exposure to primary tooth stem cells. The results indicated cell viability and the proliferation assay revealed that the clinical concentration of SDF (38%) was not cytocompatible on primary tooth stem cells. However, closer to the concentration that will diffuse toward the pulp and the dentine bridge that covers it, a concentration of 0.0038% SDF promoted cell proliferation and osteogenic differentiation. That study revealed that with the ELISA experiment, the dentine exposed to 38% SDF released TGF β-1, indicating that SDF could promote reactionary dentinogenesis (24). It is important to keep in mind that a dentine bridge of 1.5 mm is considered sufficient to limit the negative effects from eluates from dental materials, and with the carious structure above the pulp that will be remineralized with the SDF products, the possible exposure to the dental pulp also becomes more limited. In addition, the SDF products usually work as a step 1 and step 2 protocol, resulting in a more favorable result for cytocompatibility when combined (25, 26).

Stela Light Cure as a restorative material compared to Riva Light Cure presented a non-significant better cytocompatibility at both time intervals, with the 48 h time interval having a greater cytocompatibility of 57.66% compared to the 27.41% cytocompatibility of Riva Light Cure. Riva Star Aqua step 2 is more cytocompatible than Riva Star step 2. The results in Table 1 indicate that Riva Star Aqua step 2 at the 48 h interval is comparable to Stela Light cure. The cytocompatibility results therefore infer that the clinician should use their clinical judgment to prevent direct pulp contact if the healthy or carious dentine covering the pulp has a thickness of less than 1.5 mm. Riva Star, representing an SDF product in this pilot study, showed better cytocompatibility than other assessed SDF products from the literature (25). Therefore, Riva Star Aqua, from a cytocompatibility point of view, is a suitable clinical choice in addition to the other advantage it possesses over Riva Star of not containing ammonium iodide.

## 5 Conclusion

The limitation of the study was that step 1 of Riva Star and Riva Star Aqua was unable to provide a result due to the silver precipitation. In addition, mixing the two components (steps 1 and 2) of both materials and then exposing the buccal fibroblasts could have been assessed to evaluate their combined effect, but the premise of silver precipitation is still likely considering the current results. The assessment of cytocompatibility for Riva Star and Riva Star Aqua is ideal as per the dentine disc method and therefore presents a clinically relevant cytocompatibility assessment of the materials (26). The use of Stela Light Cure is a suitable material as it showed potential for increased cytocompatibility compared to Riva Light Cure.

## 6 Limitations of the study

This study is *in vitro* and is limited by not being able to capture the inherent complexity of the pulp system and its reaction to external stimulus, e.g., dental material constituents interacting with the cells. The liquids from step 1 of the Riva Star and Riva Star Aqua could not be evaluated. This is therefore a pilot study, and further research with larger sample sizes and adapted methodologies to overcome the limitations could be conducted.

## 7 Data management plan

Only the researchers have access to the data and they will not be shared with third parties. Data management practices will be compliant with the POPI Act. Data were stored securely in a durable and accessible format and in a manner that ensures its authenticity and integrity as well as meeting all legal and confidentiality requirements. The data will be retained securely within the department, in the researcher's own office, and stored on a hard drive as a password-protected file. The data might have long-term value and will be appropriately preserved and accessible for future research. Anonymized datasets will be stored for a minimum of 5 years and be deposited in the Institutional Research Data Repository on completion of the study (Kikapu data repository; https://eresearch.uwc.ac.za/kikapu/). The principal investigator will use an ORCID identifier when depositing data. Data management and storage will be done as per UWC guidelines. Research data remain the property of the university. The data management plan is therefore in line with UWC policies. Data security and management are in place on a secure Google Drive Folder and a soft copy back-up on a hard drive in a locked office at Tygerberg Oral Health Centre.

## Data Availability

The raw data supporting the conclusions of this article will be made available by the authors, without undue reservation.

## References

[1] Seifo N Robertson M MacLean J Blain K Grosse S Milne R The use of silver diamine fluoride (SDF) in dental practice. Br Dent J. (2020) 228(2):75–81. 10.1038/s41415-020-1203-9 31980777

[2] Gao SS Amarquaye G Arrow P Bansal K Bedi R Campus G Global oral health policies and guidelines: using silver diamine fluoride for caries control. Front Oral Health. (2021) 2:685557. 10.3389/froh.2021.685557 35048029 PMC8757897

[3] Kim H Lee J Lee S Kim H Park H. Evaluation of acid resistance of demineralized dentin after silver diamine fluoride and potassium iodide treatment. J Korean Acad Pediatr Dent. (2022) 49(4):392–401. 10.5933/JKAPD.2022.49.4.392

[4] Prakash M Kang YH Jain S Zandona AF. In-vitro assessment of silver diamine fluoride effect on natural carious dentin microhardness. Front Dent Med. (2021) 2:811308. 10.3389/fdmed.2021.811308

[5] Mouafy NM Ezz El Din S Shash RY Wassef N. Microhardness and bacterial inhibitory effect of Riva Star versus silver diamine fluoride on carious dentin of primary teeth (in-vitro study). Adv Dent J. (2023) 5(2):442–8. 10.21608/adjc.2023.196751.1271

[6] Ballikaya E Ünverdi GE Cehreli ZC. Management of initial carious lesions of hypomineralized molars (MIH) with silver diamine fluoride or silver-modified atraumatic restorative treatment (SMART): 1-year results of a prospective, randomized clinical trial. Clin Oral Invest. (2022) 26(2):2197–205. 10.1007/s00784-021-04236-5 PMC8572062 34743243

[7] Ribeiro APD Sacono NT Soares DG Bordini EAF de Souza Costa CA Hebling J. Human pulp response to conventional and resin-modified glass ionomer cements applied in very deep cavities. Clin Oral Investig. (2020) 24(5):1739–48. 10.1007/s00784-019-03035-3 31372829

[8] López-García S Pecci-Lloret MP Pecci-Lloret MR Oñate-Sánchez RE García-Bernal D Castelo-Baz P In vitro evaluation of the biological effects of ACTIVA kids BioACTIVE restorative, ionolux, and riva light cure on human dental pulp stem cells. Materials (Basel). (2019) 12(22):3694. 10.3390/ma12223694 31717445 PMC6888068

[9] Russo M Komatsu J Takayama S Holland Júnior C Sundfeld RH Mestrener SR Diaminofluoreto de prata. Resposta pulpar à aplicação de uma solução a 10% em dentina [Silver diamine fluoride. Pulp response to application of a 10% solution to dentin]. RGO. (1987) 35(4):264–6. . 3484047

[10] Gotjamanos T. Pulp response in primary teeth with deep residual caries treated with silver fluoride and glass ionomer cement (“atraumatic” technique). Aust Dent J. (1996) 41(5):328–34. 10.1111/j.1834-7819.1996.tb03142.x 8961607

[11] Crystal YO Marghalani AA Ureles SD Wright JT Sulyanto R Divaris K Use of silver diamine fluoride for dental caries management in children and adolescents, including those with special health care needs. Pediatr Dent. (2017) 39(5):E135–45. . 29070149

[12] Llodra JC Rodriguez A Ferrer B Menardia V Ramos T Morato M. Efficacy of silver diamine fluoride for caries reduction in primary teeth and first permanent molars of schoolchildren: 36-month clinical trial. J Dent Res. (2005) 84(8):721–4. 10.1177/154405910508400807 16040729

[13] Yamaga R Nishino M Yoshida S Yokomizo I. Diammine silver fluoride and its clinical application. J Osaka Univ Dent Sch. (1972) 12:1–20. . 4514730

[14] Kim S Nassar M Tamura Y Hiraishi N Jamleh A Nikaido T The effect of reduced glutathione on the toxicity of silver diamine fluoride in rat pulpal cells. J Appl Oral Sci. (2021) 29:e20200859. 10.1590/1678-7757-2020-0859 33886942 PMC8075293

[15] Duangthip D Wong MCM Chu CH Lo ECM. Caries arrest by topical fluorides in preschool children: 30-month results. J Dent. (2018) 70:74–9. 10.1016/j.jdent.2017.12.013 29289726

[16] Hu S Muniraj G Mishra A Hong K Lum JL Hong CHL Characterization of silver diamine fluoride cytotoxicity using microfluidic tooth-on-a-chip and gingival equivalents. Dent Mater. (2022) 38(8):1385–94. 10.1016/j.dental.2022.06.025 35778310

[17] Jackson SM Williams ML Feingold KR Elias PM. Pathobiology of the stratum corneum. West J Med. (1993) 158(3):279–85. . 8460510 PMC1311754

[18] Horst JA Ellenikiotis H Milgrom PL. UCSF Protocol for caries arrest using silver diamine fluoride: rationale, indications and consent. J Calif Dent Assoc. (2016) 44(1):16–28. . 26897901 PMC4778976

[19] Mulder R Potgieter N Noordien N. Penetration of SDF and AgF from the infected dentine towards the unaffected tooth structure. Front Oral Health. (2023) 11(4):1298211. 10.3389/froh.2023.1298211 PMC10752593 38152408

[20] Vasquez E Zegarra G Chirinos E Castillo JL Taves DR Watson GE Short term serum pharmacokinetics of diammine silver fluoride after oral application. BMC Oral Health. (2012) 12:60. 10.1186/1472-6831-12-60 23272643 PMC3538059

[21] Persaud SJ. “Cell and tissue culture: laboratory procedures in biotechnology”. In: Doyle A Griffiths JB, editors. Cell Biochemistry and Function. Hoboken, NJ: John Wiley & Sons Ltd. (1999). p. 289–332. 10.1002/(SICI)1099-0844(199912)17:4<289::AID-CBF845>3.0.CO;2-B

[22] Basak V Bahar TE Emine K Yelda K Mine K Figen S Evaluation of cytotoxicity and gelatinases activity in 3T3 fibroblast cell by root repair materials. Biotechnol Biotechnol Equip. (2016) 30:984–90. 10.1080/13102818.2016.1192960

[23] Mosmann T. Rapid colorimetric assay for cellular growth and survival: application to proliferation and cytotoxicity assays. J Immunol Methods. (1983) 65:55–6. 10.1016/0022-1759(83)90303-4 6606682

[24] Monjazebi Y. Effect of Silver Diamine Fluoride (SDF) Solution on the Stem Cells from Human Exfoliated Deciduous Teeth (SHEDs). (doctoral dissertation) (2022). Available online at: https://repository.mbru.ac.ae/handle/1/999 (accessed February 13, 2024).

[25] García-Bernal D Pecci-Lloret MP López-García S. The cytocompatibility of silver diamine fluoride on mesenchymal stromal cells from human exfoliated deciduous teeth: an in vitro study. Materials (Basel). (2022) 15:2104. 10.3390/ma15062104 35329556 PMC8954535

[26] Fernandes LDO Mendes Soares IP Anselmi C Pires MLBA Ribeiro RADO Peruchi V Pulp cell response to the application of silver diamine fluoride and potassium iodide on caries-like demineralized dentin. Clin Oral Invest. (2023):1–12. 10.1007/s00784-023-05320-8 37853265

